# Impairment of delayed recall as a predictor of amnestic mild cognitive impairment development in normal older adults: a 7-year of longitudinal cohort study in Shanghai

**DOI:** 10.1186/s12888-023-05309-3

**Published:** 2023-11-29

**Authors:** Xiaoqian Bao, Wei Li, Yuanyuan Liu, Xia Li, Ling Yue, Shifu Xiao

**Affiliations:** 1 Department of Geriatric Psychiatry, Shanghai Mental Health Center, Shanghai Jiao Tong University School of Medicine, Shanghai, 200030 China; 2 Alzheimer’s Disease and Related Disorders Center, Shanghai Jiao Tong University, Shanghai, China; 3 Shanghai Huangpu District Mental Health Center, Shanghai, China

**Keywords:** AVLT, aMCI, Longitudinal, Community, Elderly

## Abstract

**Background:**

Amnestic mild cognitive impairment (aMCI) is considered a prodromal phase of Alzheimer’s disease (AD). However, little is known about the neuropsychological characteristic at pre-MCI stage. This study aimed to investigate which neuropsychological tests could significantly predict aMCI from a seven-year longitudinal cohort study.

**Methods:**

The present study included 123 individuals with baseline cognitive normal (NC) diagnosis and a 7-year follow-up visit. All the subjects were from the China Longitudinal Aging Study (CLAS) study. Participants were divided into two groups, non-converter and converter based on whether progression to aMCI at follow-up. All participants underwent standardized comprehensive neuropsychological tests, including the Chinese Mini-Mental Status (CMMS) test, Montreal Cognitive Assessment (MoCA), auditory verbal learning test (AVLT), the digital span test, the verbal fluency test, the visual recognition test, the WAIS picture completion task, and WAIS block design. Logistic regression analysis was used to evaluate the predictive power of baseline cognitive performance for the transformation of amnestic mild cognitive impairment. Receiver operating characteristic (ROC) curve was used to test the most sensitive test for distinguishing different groups.

**Results:**

Between the non-converter group and converter group, there were significant differences in the baseline scores of AVLT-delayed recall (AVLT-DR) (8.70 ± 3.61 vs. 6.81 ± 2.96, p = 0.001) and WAIS block design (29.86 ± 7.07 vs. 26.53 ± 8.29, p = 0.041). After controlling for gender, age, and education level, converter group showed lower baseline AVLT-DR than non-converter group, while no significant difference was found in WAIS block design. Furthermore, converter group had lower AVLT-DR score after controlling for somatic disease. The area under the curve of regression equation model was 0.738 (95%CI:0.635–0.840), with a sensitivity 83.9%, specificity of 63.6%.

**Conclusions:**

Our results proved the value of delayed recall of AVLT in predicting conversion to aMCI. Early and careful checking of the cognitive function among older people should be emphasized.

## Introduction

Alzheimer’s disease (AD), a progressive neurodegenerative disorder, is the most commonly occurring dementia. However, the effectiveness of most AD treatments is limited by the late stage of AD and the intricate nature of the disorder [1]. Therefore, it is crucial to develop new diagnostic methods that can detect early-stage AD. Amnestic mild cognitive impairment (aMCI) is one of the subtypes which is most at risk for conversion to AD [2, 3]. For the early prevention, identification of individuals at pre-mild cognitive impairment (pre-MCI) stage is paramount.

It is well known that AD pathology appeared decades before the diagnosis of clinical dementia [4]. Biomarker-based tests, such as amyloid-PET or CSF studies of amyloid/tau can detect AD pathophysiological processes in vivo at preclinical stage [5–7]. However, above methods are invasive and expensive, which makes it hard to implement in a community setting. A previous study revealed that cognitive decline starts roughly 20 years before the diagnosis of incident MCI [8], indicating sensitive neuropsychological characteristics may be a potential marker at pre-MCI stage. However, compared with AD and MCI studies, neuropsychological study on pre-MCI is relatively fewer [1, 9]. For example, Pan F et al. reported a significant decrease in subtle cognitive decline compared to no cognitive impairment on the Auditory Verbal Learning Test (AVLT) [1], which implies that people may show subtle neurobehavioral changes before developing aMCI. Another study showed worse performance in visual memory and executive function in pre-MCI group [9]. However, most studies were cross-sectional [1, 9], and some of the longitudinal studies followed only two years [10], insufficient to study the prolonged AD progression. Therefore, it is necessary to study the long-term follow-up of progressive cognitive decline in normal cognitive stage and its related neuropsychological indicators.

In this study, we conducted a 7-year longitudinal community cohort study in Shanghai. A battery of neuropsychological evaluations and comprehensive clinical information, including structural magnetic resonance imaging (MRI) data were collected at baseline, and the cognition outcome were followed up. The main objective of this study was to find out which neuropsychological score best predicted the conversion of amnestic mild cognitive impairment after 7 years.

## Materials and methods

### Participants

**Fig. 1:**
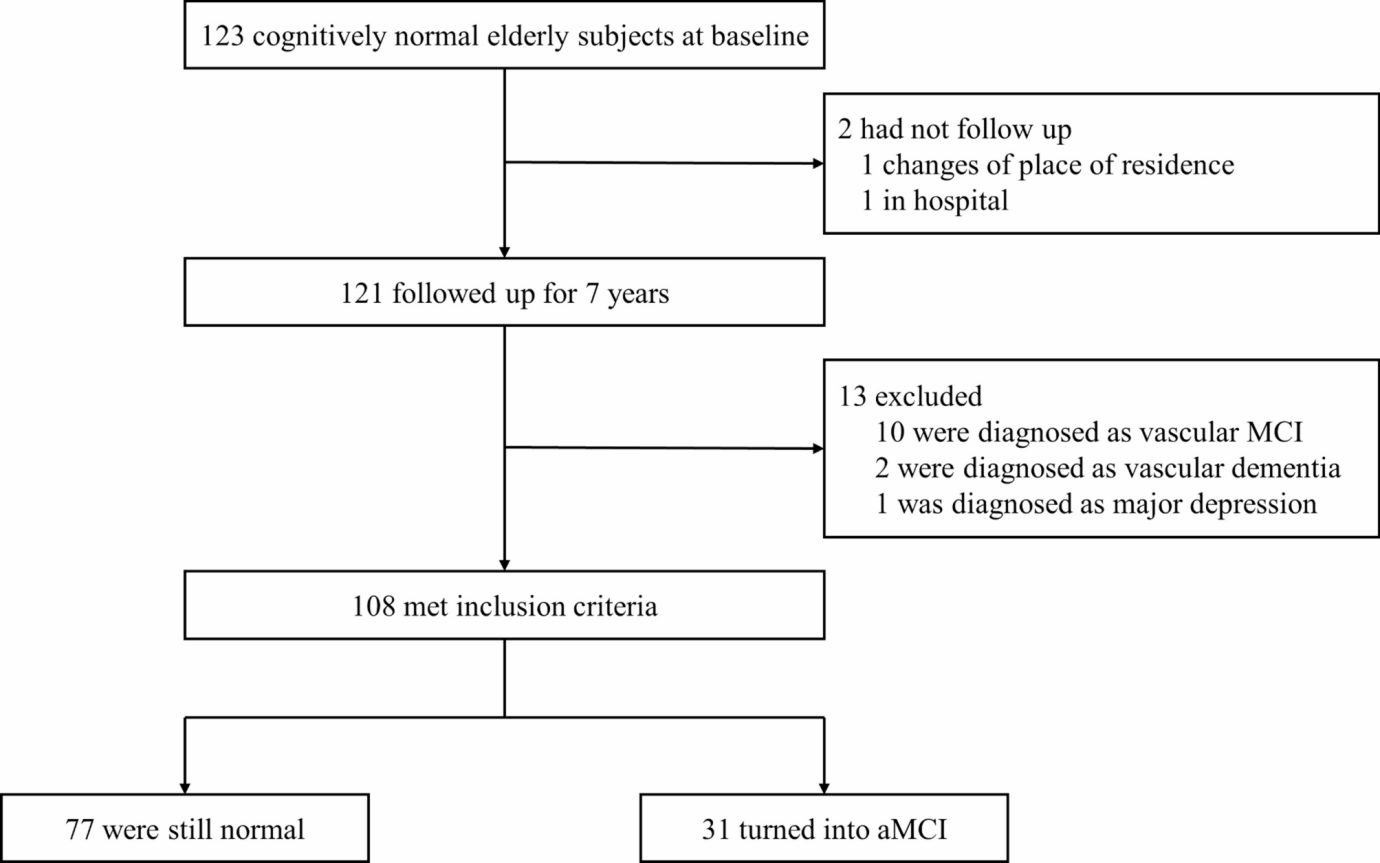
Research flow chart of the whole study

The study is a subsample of the China Longitudinal Aging Study (CLAS) [11], a community-based study of all Han Chinese aged 60 years and older in Shanghai. The project was launched in 2011. A total of 123 participants were obtained from it, who had both a baseline cognitive normal (CN) diagnosis and a 7-year follow-up visit. All of them underwent T1 cranial magnetic resonance imaging at baseline. Since the aim of the study was to predict CN to aMCI conversion, normally functioning patients who had been diagnosed with other neurodegenerative, psychological, or organic causes at follow-up were excluded. Consequently, among the 123 individuals, 15 subjects were eliminated from the analysis: two converted into vascular dementia, ten converted into vascular MCI, one converted into major depression and two were missing. At follow-up, a total of 77 participants remained stable. 31 participants transformed into aMCI. Then we grouped all aMCI cases(n = 31) into the converter group, participants who remained stable(n = 77) into the non-converter group. Figure 1 presents the research flow. aMCI was diagnosed according to the Petersen’s diagnostic criteria [12]: [1] Memory complaint usually corroborated by an informant; [2] Objective memory impairment for age; [3] Essentially preserved general cognitive function; [4] Largely intact functional activities; and [5] absence of dementia. The objective memory impairment were assessed by the recall word test in the Chinese Mini-Mental Status (CMMS) [13, 14] and the Montreal cognitive assessment (MoCA). These clinical diagnoses were made by two neuropsychologists according to accepted criteria and with consideration of comorbid conditions [15].

### Sociodemographic and health measures

Through interviews, we collected general demographic information of these participants, such as age, sex, total years of education at baseline. We also collected the information about the participants’ somatic diseases (i.e., heart disease, hypertension, diabetes mellitus, hyperlipidemia) through their self-reports and medical records at baseline.

### Neuropsychological tests

All participants were asked to complete a battery of neuropsychological tests at baseline and 7-year follow-up, including CMMS, MoCA, a Chinese version of the rey auditory-verbal learning test (AVLT), the digit span test, the verbal fluency test, the visual recognition test, the WAIS picture completion task and the WAIS block design.

The CMMS and MoCA were used to measure multiple cognitive domains including memory, attention, visual space, calculation, naming, abstraction, orientation, and language [13, 16]. The AVLT is a neuropsychological test to assess verbal memory. In the AVLT test, the rater reads out 15 nouns, with intervals of one second between each word. The subject is asked to recall these words. After this first immediate free recall trial, there are four free recall trials. The total scores for these five trials are recorded as AVLT immediate recall (AVLT-IR). After a 20-minute delay period, the subject is asked to recall as many words as possible from memory again. The number of words that were recalled in this trial is recorded as AVLT delayed recall (AVLT-DR) [17]. The digit span test is a measure of working memory and verbal short term, including forward digit span and reverse digit span [18]. The verbal fluency test is widely used to detect executive dysfunction. The participant is asked to generate words from initial letters in 60 s [19]. The visual recognition test ​is that requires participants to recognize a series of familiar objects during a visual presentation of a single or multiple objects [18]. The WAIS picture completion task measures visual perception and organization, attention, and visual recognition of basic details of objects15). The WAIS block design is used to assess motor and executive function [20].

The neuropsychological tests for each visit were performed by the same team of trained neuropsychologists following a standard protocol.

### Magnetic resonance imaging acquisition and processing

All participants were scanned on a Siemens Magnetom Verio 3.0T MRI scanner (Siemens, Munich, Germen) at baseline [21]. The parameters of T1- weighted 3D magnetization prepared rapid gradient echo (MPRAGE) sequences were as follows: TR = 2,300 ms, TE = 2.98 ms, flip angle of 9 degrees; matrix size = 240 × 256; field of view (FOV) = 240 × 256 mm; slice thickness = 1.2 mm. Automated procedure which has been described by Wolz et al. [22] was used to assess volumetric data. Memory complaints are associated with volume reduction of hippocampus and amygdala [21, 23, 24]. For each subject, volume with hippocampus and amygdala were extracted.

### Statistical analysis

Continuous variables were shown as mean ± standard deviation (SD), categorical variables were shown as frequencies (%). The Shapiro-Wilk test was used to check whether the data were normal distribution. The independent samples t-test and Mann-Whitney U test were used to compare the demographic data, brain volume and neuropsychological tests scores between the non-converter group and converter group. Categorical variables were compared by Chi-square analysis. In order to address possible confounding effects, the neuropsychological tests scores were analyzed by binary logistic regression in three models. Neuropsychological tests scores were used as explanatory variables and the group as dependent variable in Model 1. ​As previously reported, the incidence of aMCI varies widely by age, sex, and education level [25]. Therefore, in Model 2, gender, age and education level (high education: years of education >12; secondary education: years of education = 7–12; primary education: years of education ≤ 6), were controlled as covariates. Furthermore, heart disease, hypertension, diabetes mellitus and hyperlipidemia were considered to be risk factors accounting for AD [26, 27]. We controlled them as covariates additionally in Model 3. Receiver Operating Characteristic (ROC) curve was used to assess the sensitivity and specificity of different models to predict aMCI. The data was analyzed by SPSS 23.0. The significance level was set at p<0.05.

## Results

### General demographic data and brain volume

The demographic and brain volume of the participants are presented in Table 1. After 7 years, 31 participants (29%) had converted to aMCI. The groups were similar in age and gender. No statistical difference was found between the groups in somatic disease. Compared to non-converter, the participants in the converter group were less educated (9.97 ± 3.23 vs. 7.90 ± 3.59, year, p = 0.006). No statistical difference in hippocampal and amygdala volumes was found between non-converter and converter.

**Table 1:** Demography, brain volume and neuropsychological test between non-converter and converter at baseline (N = 108)

	Non-converter (N = 77)	Converter (N = 31)	F/z/χ^2^	*p*
Age (year)	68.00 ± 7.25	69.74 ± 6.27	−1.658	0.970
Education (year)	9.97 ± 3.23	7.90 ± 3.59	−2.772	0.006*
Male, n (%)	38 (49.3%)	15 (48.4%)	0.008	1.000
CMMS	28.26 ± 1.61	27.61 ± 1.94	−1.556	0.120
MoCA	25.27 ± 3.50	24.03 ± 3.88	−1.684	0.092
Digit span				
Forward	9.69 ± 2.27	9.42 ± 2.23	−0.627	0.531
Backward	5.94 ± 2.29	5.68 ± 2.12	−0.487	0.626
AVLT				
AVLT-IR	33.64 ± 8.99	31.84 ± 10.84	2.441	0.378
AVLT-DR	8.70 ± 3.61	6.81 ± 2.96	−3.305	0.001*
Verbal fluency average^a^	9.886 ± 2.99	9.247 ± 3.56	−1.083	0.279
Visual recognition				
Functional^a^	3.66 ± 0.62	3.52 ± 0.68	−1.158	0.247
Semantic^a^	3.32 ± 0.79	3.26 ± 1.00	−0.102	0.919
Correct^b^	6.13 ± 1.30	6.07 ± 1.44	−0.054	0.957
WAIS picture completion	11.60 ± 4.07	10.29 ± 4.24	−1.210	0.226
WAIS block design^b^	29.86 ± 7.07	26.53 ± 8.29	0.096	0.041*
Left hippocampus	3664.61 ± 416.75	3552.50 ± 450.77	0.002	0.219
Right hippocampus	3834.27 ± 526.41	3822.51 ± 409.46	−0.431	0.666
Left amygdala	1530.66 ± 215.46	1475.03 ± 254.06	0.289	0.592
Right amygdala	1651.25 ± 247.76	1681.33 ± 224.88	−0.438	0.661
Heart disease, n (%)	16 (20.8%)	8 (25.8%)	0.323	0.613
Hypertension, n (%)	36 (46.8%)	16 (51.6%)	0.209	0.676
Diabetes mellitus, n (%)	10 (13.0%)	5 (16.1%)	0.182	0.760
Hyperlipidemia, n (%)	9 (11.7%)	4 (12.9%)	0.031	1.000

*AVLT-IR* Auditory Verbal Learning Test immediate recall, *AVLT-DR* Auditory Verbal Learning Test delayed recall, *CMMS* Chinese Mini-Mental Status, *MoCA* Montreal cognitive assessment, *WAIS* Wechsler Adult Intelligence Scale

^*^p < 0.05

^a^One sample data was missing

^b^Two sample data were missing

### Neuropsychological tests

The non-converter group showed higher baseline AVLT-DR (8.70 ± 3.61 vs. 6.81 ± 2.96, p = 0.001) and WAIS block design score (29.86 ± 7.07 vs. 26.53 ± 8.29, p = 0.041) than the converter group, while no statistical difference (p > 0.05) was found in CMMS, MoCA, the digital span test, the verbal fluency test, the visual recognition test, the visual matching and reasoning, WAIS picture completion task and total scores of AVLT (AVLT-IR) (Table 1). After 7-year follow-up, all the neuropsychological test scores except the correct answer of visual recognition in the converter group were lower than the non-converter group (Table 2).

**Table 2:** Demography, brain volume and neuropsychological test between non-converter and converter after 7-year follow-up (N = 108)

	Non-converter (N = 77)	Converter (N = 31)	F/z/χ^2^	*p*
CMMS	27.61 ± 1.84	24.71 ± 3.12	−4.655	0.000**
MoCA	23.21 ± 3.66	17.87 ± 3.70	−5.537	0.000**
Digit span				
Forward	8.42 ± 2.37	7.19 ± 2.36	−2.273	0.023*
Backward	5.29 ± 1.94	4.16 ± 1.29	−2.790	0.005*
AVLT				
AVLT-IR	32.05 ± 8.92	22.94 ± 9.31	−5.040	0.000**
AVLT-DR	9.55 ± 3.67	4.97 ± 4.36	−4.631	0.000**
Verbal fluency average^a^	8.79 ± 2.32	6.84 ± 2.23	−3.426	0.001*
Visual recognition				
Functional^a^	3.62 ± 0.63	3.06 ± 0.93	−3.518	0.000**
Semantic^a^	3.12 ± 0.76	2.45 ± 1.18	−2.792	0.005*
Correct^b^	5.65 ± 1.64	5.23 ± 2.00	−0.970	0.332
WAIS picture completion	10.69 ± 3.25	8.77 ± 3.29	0.025	0.007*
WAIS block design^b^	29.86 ± 7.07	26.53 ± 8.29	−4.170	0.000**

*AVLT-IR* Auditory Verbal Learning Test immediate recall, *AVLT-DR* Auditory Verbal Learning Test delayed recall, *CMMS* Chinese Mini-Mental Status, *MoCA* Montreal cognitive assessment, *WAIS* Wechsler Adult Intelligence Scale

^*^p < 0.05; **p < 0.001

^a^One sample data was missing

^b^Two sample data were missing

Logistic regression was used to evaluate the association between these neuropsychological tests and different diagnosis groups in two models to address possible confounding effects (Table 3). Significant differences were found between the converter group and non-converter group in baseline AVLT-DR score for model1 (OR = 0.853, p = 0.014) and model2 (OR = 0.870, p = 0.042). Significant difference was found between two groups in WAIS block design for model1 (OR = 0.942, p = 0.045). However, no significant difference was found for model2 (p = 0.402) in it.

**Table 3:** Binary logistic regressions among AVLT-DR and WAIS block scores at baseline

		OR (95%CI)	p
AVLT-DR	Model 1	0.853 (0.751–0.968)	0.042*
	Model 2	0.870 (0.760–0.995)	0.042*
WAIS block	Model 1	0.942 (0.888–0.999)	0.045*
	Model 2	/	0.402

Note: AVLT-DR = Auditory Verbal Learning Test delayed recall; WAIS = Wechsler Adult Intelligence Scale; *p<0.05

Model 1 used AVLT-DR and WAIS block as explanatory variables; Model 2 was adjusted for gender, age, and education level

Since AVLT-DR was the only neuropsychological test that could distinguish aMCI converters from non-converters, we added more covariates (i.e., heart disease, hypertension, diabetes mellitus, hyperlipidemia) into binary stepwise logistic regression analysis to build model3. Table 4 shows the result. Significant difference was also found in baseline AVLT-DR score (OR = 0.857, p = 0.033). The possibility of aMCI conversion from normal cognitive function increased with a lower baseline AVLT-DR score.

**Table 4:** Binary logistic regressions analysis of model 3

	B	S.E.	*p*	OR	95%CI	
					Lower	Upper
AVLT-DR	-0.154	0.072	0.033*	0.857	0.744	0.988
Education level	/	/	0.165	/	/	/
Gender(male/female)	/	/	0.784	/	/	/
Age(year)	/	/	0.626	/	/	/
Hypertension	/	/	0.835	/	/	/
Heart disease	/	/	0.597	/	/	/
Diabetes	/	/	0.710	/	/	/
Hyperlipidemia	/	/	0.372	/	/	/

Note: AVLT-DR = Auditory Verbal Learning Test delayed recall; *p<0.05

Model 3 was additionally adjusted for heart disease, hypertension, diabetes mellitus, hyperlipidemia

### Receiver operating characteristic (ROC) curves

The ROC curve was used to estimate the sensitivity and specificity for baseline AVLT-DR, WAIS block design and model3. Table 5 showed the result. Model3 had the largest area under the curve (0.738, p = 0.000, 95%CI:0.635–0.840). Moreover, the result showed that Model 3 had a moderate effect on predicting aMCI. The sensitivity was 83.9% and the specificity was 63.6%.

**Table 5:** ROC analysis of baseline AVLT-DR scores, WAIS block scores and Model3 for differentiating converters and non-converter

	AUC	Sensitivity	Specificity	OR (95%CI)	p
AVLT-DR	0.690	0.677	0.636	0.581–0.799	0.002*
WAIS block	0.623	0.867	0.342	0.506–0.740	0.049*
Model3	0.738	0.839	0.636	0.635–0.840	0.000**

Note: ROC = Receiver Operating Characteristic; AUC = area under the curve; AVLT-DR = Auditory Verbal Learning Test delayed recall; WAIS = Wechsler Adult Intelligence Scale; *p<0.05; **p<0.001

## Discussion

This study investigated baseline performance on neuropsychological tests among 108 elderly people with normal cognitive function in Shanghai. The main finding was that the AVLT-DR provided the best value in distinguishing the converter group and the non-converter group. The baseline AVLT-DR score was significantly lower in the converter group than in the non-converter group. It was a predictor of aMCI-converter with a high sensitivity. The baseline WAIS block design score also was lower in the converter group. However, after adjustment by gender, age, and education level, no significant difference was found between the groups. Other baseline neuropsychological tests did not differ between the groups. No significant difference was found in volume of hippocampus and amygdala in the converter group and non-converter group.

Weak memory performance was found to be a sensitive predictor of MCI to dementia in many studies [8, 28, 29]. It was reported that episodic memory decline is a symptom of early AD [30]. AVLT is widely used to assess the episodic memory. In our study, it showed strong value in aMCI conversion from normal cognition. No significant difference was found in CMMS, MoCA, and the other neuropsychological tests between the groups at baseline in our study. AVLT has a better assessment of delayed recall when compared to CMMS and MoCA. In a 2-year follow-up, AVLT-DR was reported to be a significant predictor of AD conversion, while AVLT-IR was not [30]. Likewise, no significant difference was found in AVLT-IR between the non-converter group and converter group in our study. The result was fit well with previous findings that delayed recall decline was one of the most sensitive symptoms for early diagnosis of AD [29, 31].

After adjustment for gender, age, and education level, the baseline WAIS block design scores did not differ between the converter group and the non-converter group. It could be explained by the impact of education on cognition. The education level was found to be positively correlated with WAIS block design performance, such people with less year of education performed worse on it [32].

Structural MRI of medial temporal atrophy (MTA) is considered a biomarker for early diagnosis of AD and MCI [33, 34]. The volume reduction of medial temporal lobe, including hippocampus and amygdala is a very early manifestation of AD [24, 34]. The medial temporal regions are critical for episodic memory [35]. However, no significant difference in volume of hippocampus and amygdala was found between the groups in this study. The difference between our results and the previous studies could be explained by the reason that the delayed recall impairment happened before MTA change. Resting-state functional MRI can be used to further explore the participant’s medial temporal lobe.

This study has some limitations. First, since the sample size is relatively small, further validation is needed. Secondly, although the area under the ROC curve of the Model 3 was 0.738 and the sensitivity was 83.9%, the specificity was 63.6%. AVLT is still not enough on its own to accurately predict the occurrence of aMCI. So, it needs to be combined with other tests such as biomarkers for more accurate predictions. Next, PET, amyloid markers and APOE genotype associated with AD were not tested in this study. Correlation analysis of AVLT-DR with these biomarkers could not be performed. The correlation between AVLT and them can be further explored.

## Conclusions

In conclusion, the baseline AVLT-DR score is negatively associated with the occurrence of aMCI through a 7-year follow-up study in the community. AVLT-DR is a sensitive predictor of NC-to-aMCI conversion. In future studies, we will further compare the sensitivity and specificity of AVLT, PET, amyloid markers, APOE genotype and other biomarkers in predicting aMCI.

## Data Availability

The datasets used and/or analyzed during the current study are available from the corresponding author on reasonable request.
